# *In silico *identification of novel lead compounds with AT_1 _receptor antagonist activity: successful application of chemical database screening protocol

**DOI:** 10.1186/2191-2858-2-7

**Published:** 2012-03-01

**Authors:** Mahima Pal, Sarvesh Paliwal

**Affiliations:** 1 Department of Pharmacy, Banasthali University, Banasthali, Tonk, Rajasthan, India

**Keywords:** angiotensin II receptor antagonists, *N*^2^-aryl biphenyl triazolinone, pharmacophore mapping

## Abstract

**Background:**

AT_1 _receptor antagonists are clinically effective drugs for the treatment of hypertension, cardiovascular, and related disorders. In an attempt to identify new AT_1 _receptor antagonists, a pharmacophore-based virtual screening protocol was applied. The pharmacophore models were generated from 30 training set compounds. The best model was chosen on the basis of squared correlation coefficient of training set and internal test set. The validity of the developed model was also ensured using catScramble validation method and external test set prediction.

**Results:**

The final model highlighted the importance of hydrogen bond acceptor, hydrophobic aliphatic, hydrophobic, and ring aromatic features. The model satisfied all the statistical criteria such as cost function analysis and correlation coefficient. The result of estimated activity for internal and external test set compounds reveals that the generated model has high prediction capability. The validated pharmacophore model was further used for mining of 56000 compound database (MiniMaybridge). Total 141 hits were obtained and all the hits were checked for druggability, this led to the identification of two active druggable AT_1 _receptor antagonists with diverse structure.

**Conclusion:**

A highly validated pharmacophore model generated in this study identified two novel druggable AT_1 _receptor antagonists. The developed model can also be further used for mining of other virtual database.

## 1. Background

The renin-angiotensin system plays a fundamental role in blood pressure and fluid and electrolyte homeostasis [1]. Angiotensin II (AII), an octapeptide produced by the renin-angiotensin system, is a powerful endogenous vasopressor. Angiotensin converting enzyme inhibitors work by blocking the production of angiotensin II from angiotensin I. An alternative and possibly superior approach would be to block the action of AII at the level of its receptor. Two distinct subtypes of AII receptors [type 1 (AT_1_) and type 2 (AT2)] have been identified, and both belong to the G protein-coupled receptors super family (GPCRs) [2,3]. Most of the biological actions of AII are mediated by the AII receptors of the AT_1 _subtype. The AT_1 _receptor subtype mediates virtually all the known physiological actions of AII in cardiovascular, neuronal, endocrine, and hepatic cells as well as in other ones. Since AT_1 _receptor is GPCR the interaction of AII with the AT_1 _receptor induces a conformational change, which promotes the coupling with the G protein(s) and leads to the signal transduction via several effector systems (phospholipases C, D, A2, adenyl cyclase, etc.). The AT_1 _receptors play a major role in the pressor and trophic actions of the AII, and much effort has been spent in developing nonpeptide antagonists for this receptor for the treatment of hypertension and congestive heart failure [4].

Like other GPCR families, AT_1 _receptors are transmembrane proteins and such macromolecules are not easily crystallized for structural analysis by X-ray crystallography [5]. In the absence of three-dimensional (3D) structure for AT_1 _receptor, a rational design of antagonists using a structure-based approach is not feasible [1]. For this reason, 3D pharmacophore models from the ligand-based approach are very useful for analyzing the ligand-receptor interactions. Moreover, a pharmacophore can also be used as a query in a 3D database search to identify new structural classes of potential lead compounds. In the recent years, the development of a 3D-pharmacophore and its use in the virtual screening of the chemical databases appear to be a more relevant and time-saving approach. Thus, the construction of an accurate pharmacophore is a key objective in many drug discovery efforts.

The pharmacophore generation methods of the Catalyst software have been successfully used in drug discovery research and toxicology [6-8] as evident from pharmacophore-based development of protein farnesyl transferase, human immunodeficiency virus (HIV) protease, and HIV reverse transcriptase inhibitors [9,10].

In this study, our approach of pharmacophoric exploration via set of diverse 3D structures has resulted in development of a highly validated and predictive pharmacophore model for AT_1 _receptor antagonists. The developed phamacophore was subsequently used for virtual screening of chemical databases for identification of novel lead compounds with nanomolar activity range.

## 2. Results and discussion

### 2.1. HypoGen model

Pharmacophore models were generated using 30 training set compounds representing two series of structurally diverse compounds with AT_1 _receptor antagonist activity. All the generated pharmacophore hypotheses were evaluated for their statistical fitness on the basis cost difference values, correlation coefficients (*r*), and rms deviations. The pharmacophoric features and statistical data for a set of ten chosen hypothesis are listed in Additional file 1.

Out of ten, hypothesis1 was identified as best pharmacophore model, since this hypothesis showed a cost difference of 20.17 between null cost 148.75 and total cost 128.58 satisfying the range recommended in the cost analysis of the catalyst procedure. Hypothesis1 had total cost close to fixed cost (124.52), lower error cost (103.409), lowest root-mean-square (RMS) divergence (0.408), best correlation (*r *= 0.977), and good internal test set prediction (*r*_test-set _= 0.93). The configuration cost of the hypothesis exceeded the limit of 17 bits but can be accepted as the model achieves other validation criterion [11,12].

The chosen hypothesis comprised of one hydrogen-bond acceptor (HBA), hydrophobic aliphatic region, and hydrophobic (HY) and one ring aromatic (RA) sites in a specific 3D orientation. The results of tolerance and weight fit to the features of the training set compounds are given in Additional file 2. The pharmacophore model mapped well to the training and test set compounds. The values of actual and predicted activity for the training and internal test set compounds are given in Additional files 3 and 4. The model was found to be quite good in predicting the activity of external test set compounds [13] with correlation co-efficient value of 0.71 and the values of actual and predicted activity are given in Additional file 5.

### 2.2. Fisher's cross validation test

**Figure 1:**
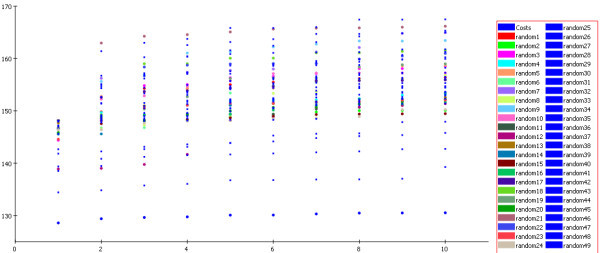
**Graph of the Cat-scrambled data generated from training set**.

The Fisher's randomization test was used to validate the strong correlation between chemical structures and biological activity. The generated pharmacophore model was assessed for quality by Fischer randomization test method using Cat Scramble technique in Catalyst at 98% confidence. The results are shown in Figure 1 and the resultant data clearly shows that none of the outcome hypothesis had a lower cost score than the initial hypothesis. The results obtained clearly supported the validity of selected pharmacophore model.

### 2.3. Mapping of training set compounds

**Figure 2:**
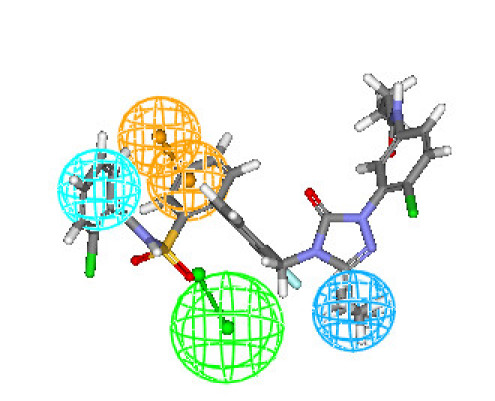
**Best conformation of compound 6b fit to the generated pharmacophore model of AT_1 _receptor antagonists**.

Hypothesis1 is presented in Figure 2, aligned with the most active compound (**6b**: 0.072 nM) of the training set molecules. For this compound, HBA feature mapped to the S = O group of sulfonamide moiety. The HY aliphatic group mapped to the butyl chain at the triazolinone ring and the other HY feature mapped to the chlorophenyl ring. Ring aromatic feature mapped to one of the phenyl ring of biphenyl ring.

**Figure 3:**
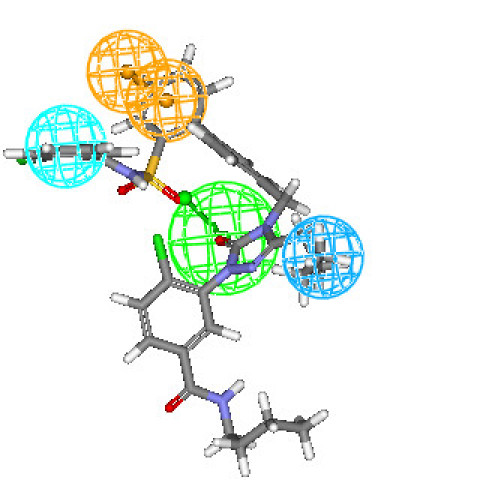
**Best conformation of compound 7a fit to the generated pharmacophore model of AT_1 _receptor antagonists**.

**Figure 4:**
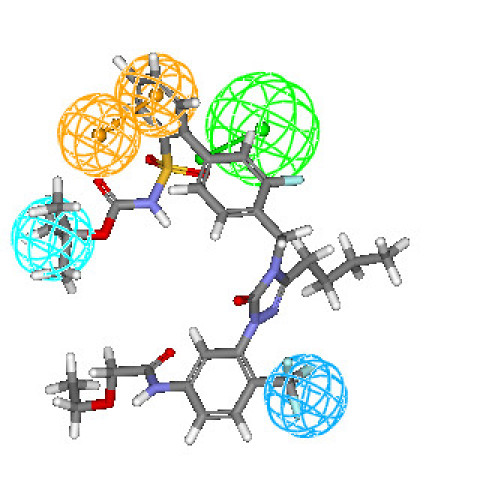
**Best conformation of compound 16 fit to the generated pharmacophore model of AT_1 _receptor antagonists**.

Figures 3 and 4 depict one of the conformations of compounds **7a **and **16 **in the training set mapped onto Hypothesis1. As seen from these figures, both the compound fit all features of the developed pharmacophore model very well similar to the most active compound. Moreover, compounds **7a **and **16 **were reasonably well estimated with a fit value of 9.42 and 9.15, respectively (actual activity (**7a**) 0.14 nM; estimated 0.13 nM and actual activity (**16**) 0.26 nM; estimated 0.251 nM).

**Figure 5:**
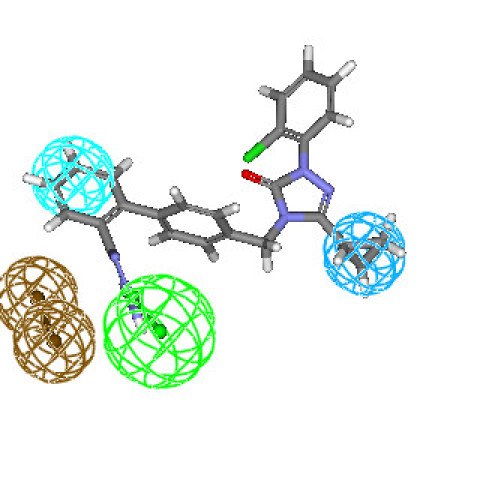
**Best conformation of compound 33e fit to the generated pharmacophore model of AT_1 _receptor antagonists**.

The most active compounds in the dataset assumed conformations that allowed proper mapping of all the feature of the generated hypotheses, whereas least active compounds were unable to map HY aliphatic or ring aromatic. Pharmacophore mapping of the least active compound **33e **is shown in Figure 5.

### 2.4. Mapping of test set compounds

**Figure 6:**
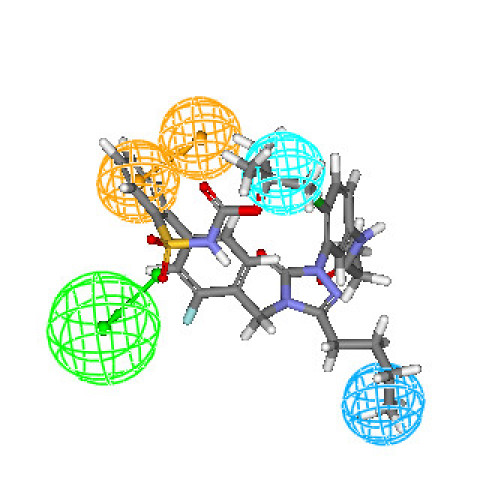
**Best conformation of compound 5b fit to the generated pharmacophore model of AT_1 _receptor antagonists**.

**Figure 7:**
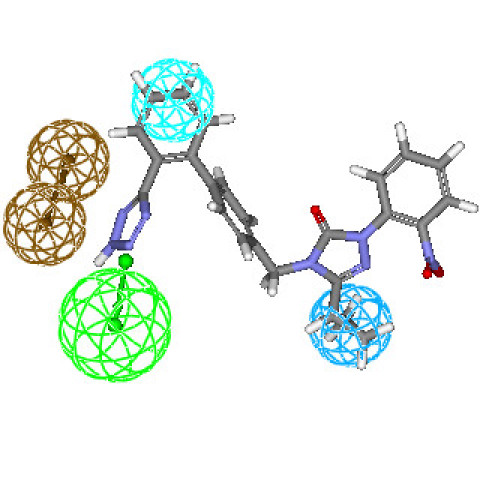
**Best conformation of compound 38e fit to the generated pharmacophore model of AT_1 _receptor antagonists**.

Hypothesis1 was further studied for its mapping pattern for the compounds of test set. The mapping analysis of the compounds, namely **5b **in the test set, revealed that none of the essential pharmacophoric features were missed and all features mapped with the least displacement from the centroid of all features (Figure 6). The t-butyl group of **5b **mapped well with the HY feature of the pharmacophoric model, and the butyl group mapped with HY feature. The oxygen of the SO_2 _mapped to the HBA feature, while the ring aromatic feature mapped to the phenyl ring of the biphenyl ring groups of compound **5b**. The moderate and lesser active compounds missed to map one pharmacophoric features and thereby justifying their corresponding categories. The moderately active compounds **35e **and **36e **missed the HY feature while the lesser active compound **38e **(Figure 7) missed the ring aromatic feature. These results revealed the importance of HY functionalities and ring aromatic feature in imparting good AT1 receptor antagonist activity.

### 2.5. Database search

The validated pharmacophore model was used to search MiniMaybridge and NCI chemical databases [14,15] for identification of new AT_1 _antagonists. By employing the fast search algorithm, 141 hits were retrieved. Subsequently, the hits were subjected to additional filtering to exclude compounds with low potency and unfavorable absorption and permeation properties. This led to the repossession of five structurally diverse druggable compounds with nanomolar activities (Table 1).

**Table 1:** List of hits obtained from the MiniMaybridge and NCI database with their corresponding fit value and estimated activity

Number	Hits retrieved	Fit value	Estimated activity (nM)
1 (MiniMaybridge HITS)	SP 01066	9.239	0.205
2	KM 09509	8.524	1.062
3(NCI HITS)	NSC 122371	9.517	0.108
4	NSC 157629	9.515	0.108
5	NSC 90956	9.356	0.156

## 3. Materials and methods

*Disco*v*ery studio*, version 2.0, Accelrys Software Inc., San Diego, CA, was used to develop pharmacophore hypothesis for structurally diverse series of triazolinone derivatives reported in the literature [16,17] with activity range from 0.072 to 250 nM. Chemical structures of various *N*^2^-aryltriazolinone biphenylsulfonamides with their experimental IC_50 _values for the AT_1 _receptor subtype are listed in Additional file 6.

### 3.1. Selection of the training set and test set

The most important aspect of the hypothesis generation in HypoGen is the selection of the training set of molecules. The selection has to follow some basic requirements; such as a minimum of 16 structurally diverse compounds should be selected to avoid any chance correlation, most active compound should be included and the activity data should have a range of 3.5-5 orders of magnitude [18].

On the basis of above criteria, the dataset was divided into training set and test set. The training set comprised of 30 compounds, whereas internal test set was composed of 27 compounds. The most active compounds were included in the training set so that they would provide critical information for pharmacophore requirements. Several moderately active and inactive compounds were also included to spread the activity ranges as wide as possible. The important aspect of such selection scheme is that each active compound should teach something new to the HypoGen module to help it uncover as much critical information as possible for predicting biological activity.

### 3.2. Generation of pharmacophores

Details of the pharmacophore development procedures have been described in the literature [9,18]. In brief, conformational models of all training set molecules with AT_1 _receptor antagonist activity were generated using the best quality conformational search option in Catalyst employing a constraint of a 20 kcal/mol energy threshold above the global energy minimum using CHARMm force field. A maximum of 250 conformations were generated to ensure maximum coverage in the conformational space [19]. Instead of using just the lowest energy conformation of each compound, all conformational models for molecules in each training set were used in for pharmacophore hypothesis generation. The Catalyst software can generate pharmacophore hypotheses consisting of a maximum of five features. An initial analysis revealed that four chemical feature types such as HBA, HY, hydrophobic aliphatic (HY-ALI), and ring aromatic (RA) could effectively map all critical chemical features of all molecules in the training set. The minimum and maximum counts for HBA, HY, HY-ALI, and RA were set to 0 and 3, respectively. These four feature types were used to generate ten pharmacophore hypothesis from the training set. The uncertainty value was defaulted to 3 which is a ratio range of uncertainty in the activity value and MinPoints and MinSubset-Points were 4 (default value). The MinPoints parameter controls the minimum number of location constraints required for any hypothesis. The MinSubsetPoint parameter defines the number of chemical features that a hypothesis must match in all the compounds set [20].

### 3.3. Evaluation of the HypoGen model

#### 3.3.1. Cost function analysis

All the hypotheses generated were subjected to cost function analysis which is considered as stringent quality check tool. Two important theoretical cost calculations that determine the success of any pharmacophore hypothesis are "fixed cost" and "null cost". Fixed cost represents the simplest model that fits all data perfectly, and the second null cost represents the highest cost of a pharmacophore with no features and which estimates activity to be the average of the activity data of the training set molecules. The null cost value is equal to the maximum occurring error cost. The greater the difference between null cost and total cost and closer the total cost of the generated hypothesis to the fixed cost, the more statistically significant is the generated hypothesis. Another important cost is the overall cost of HypoGen model which consist of three cost components, the weight cost, the error cost, and the configuration cost. The quality of each hypothesis can be judged on the basis of total cost which is sum of error cost, weight cost, and configuration cost. The configuration cost, which is also known as the entropy cost, depends on the complexity of the pharmacophore hypothesis space. The error cost is dependent on the RMS differences between the estimated and the actual activities of the training set molecules. In standard HypoGen model, the configuration should not be greater than 17.0. The RMS deviations represent the quality of the correlation between the estimated and the actual activity data. The error cost is the most important part of the total cost and increases as the RMS difference between the estimated and the actual affinity for the training set increases. The RMS value is related to the quality of prediction of the hypothesis. Error cost provides the highest contribution to total cost and it is directly related to the capacity of the particular pharmacophore as 3D QSAR model, i.e., in correlating the molecular structures to the corresponding biological responses. The weight cost is a value that increases in a gaussian form as the difference between the actual and ideal weights of the features deviates. According to the documentation, the ideal value of the weight is 2 because higher weight values tend to force unrealistic conformations of the compounds to fit such features [20].

#### 3.3.2. Test set prediction

The ability of the models to predict the biological activity of compounds outside the model development procedure is a common method of validation [21]. Internal test set of 27 and external test set of 46 compounds were employed to assess statistical significance of the developed model. All test set molecules were built and minimized as well as used in conformational analysis like the training set molecules. Predictions were made to evaluate the level of similarity between actual and predicted activity.

#### 3.3.3. Statistical validation

Statistical cross-validation study was performed to assess the significance of the best hypotheses using the catScramble program available in Catalyst. The statistical significance is given by the equation.

Significance=[1−(1+x)/y]×10

where *x *is the total number of hypotheses having a total cost lower than best significant hypothesis and *y *the number (HypoGen runs initial + random runs). To obtain a 95% confidence level, 19 random spreadsheets are generated (*y *= 20) and every generated spreadsheet is submitted to HypoGen using the same experimental conditions (functions and parameters) as the initial run.

## 4. Database mining

The generated validated pharmacophore was used as query to search the virtual chemical compound database (NCI and MiniMaybridge) to identify new lead compounds with AT_1 _receptor antagonist activity.

## 5. Conclusion

The quantitative pharmacophore models were developed using the training set of molecules with the help of HypoGen module implemented in the Catalyst. The best pharmacophore model provided a statistically significant correlation and well-estimated AT_1 _activities for the test set compounds. Pharmacophore models generated for AT_1 _antagonists in this study highlight the structural requirements for antagonistic activity. This study also helped in the identification of five structurally diverse AT_1 _receptor antagonists.

## Competing interests

The authors declare that they have no competing interests.

## Supplementary Material

Additional file 1 **Pharmacophoric hypotheses generated with training set of molecules using the HypoGen algorithm**. The file contains the details of the generated pharmacophore models using two series of structurally diverse compounds with AT_1 _receptor antagonist activity alongwith their statistical fitness on the basis of cost difference values, correlation coefficients (*r*), and rms deviations. Click here for file

Additional file 2 **Pharmacophoric features and corresponding weights, tolerances, and 3D coordinates of best model**. The file contains the details of the features retrieved (hydrogen-bond acceptor, hydrophobic aliphatic, hydrophobic, and ring aromatic) and the tolerance and weight fit to the features of the training set compounds. Click here for file

Additional file 3 **Actual versus estimated activity and the selected chemical features of the final pharmacophoric model for training set of compounds**. The file contains the comparison of the estimated and actual activity along with feature mapping status for training set of compounds. Click here for file

Additional file 4 **Actual versus estimated activity and the selected chemical features of the final pharmacophoric model for internal test set of compounds**. The file contains the comparison of the estimated and actual activity along with feature mapping status for internal test set of compounds. Click here for file

Additional file 5 **Actual versus estimated activity and the selected chemical features of the final pharmacophoric model for external test set of compounds**. The file contains the comparison of the estimated and actual activity along with feature mapping status for external test set of compounds. Click here for file

Additional file 6 **Chemical structures of various *N*^2^-aryltriazolinone biphenylsulfonamides with their experimental IC_50 _values for the AT_1 _receptor subtype**. The file contains the structural and activity details of the series of the compound used in present study. Click here for file
